# Gadoxetic acid–enhanced magnetic resonance imaging to predict paritaprevir-induced hyperbilirubinemia during treatment of hepatitis C

**DOI:** 10.1371/journal.pone.0196747

**Published:** 2018-04-30

**Authors:** Hironao Okubo, Hitoshi Ando, Yushi Sorin, Eisuke Nakadera, Hiroo Fukada, Junichi Morishige, Akihisa Miyazaki, Kenichi Ikejima

**Affiliations:** 1 Department of Gastroenterology, Juntendo University Nerima Hospital, Tokyo, Japan; 2 Department of Cellular and Molecular Function Analysis, Kanazawa University Graduate School of Medical Sciences, Ishikawa, Japan; 3 Department of Gastroenterology, Juntendo University School of Medicine, Tokyo, Japan; Hokkaido University, JAPAN

## Abstract

**Background:**

Paritaprevir inhibits organic anion–transporting polypeptide (OATP)1B1 and OATP1B3, which transport bilirubin. Hyperbilirubinemia is an adverse event reported during hepatitis C treatment. Gadoxetic acid is also transported by OATP1B1/1B3. We evaluated whether the enhancement effect in gadoxetic acid–enhanced magnetic resonance (MR) imaging could predict the plasma concentration of paritaprevir and might anticipate the development of hyperbilirubinemia.

**Methods:**

This prospective study evaluated 27 patients with hepatitis C who underwent gadoxetic acid–enhanced MR imaging prior to treatment with ombitasvir, paritaprevir, and ritonavir. The contrast enhancement index (CEI), a measure of liver enhancement during the hepatobiliary phase, was assessed. Plasma trough concentrations, and concentrations at 2, 4, and 6 h after dosing were determined 7 d after the start of treatment.

**Results:**

Seven patients (26%) developed hyperbilirubinemia (≥ 1.6 mg/dl). Paritaprevir trough concentration (C_trough_) was significantly higher in patients with hyperbilirubinemia than in those without (*p* = 0.022). We found an inverse relationship between CEI and C_trough_ (r = 0.612, *p* = 0.001), while there was not a significantly weak inverse relationship between AUC_0–6 h_ and CEI (r = −0.338, *p* = 0.085). The partial correlation coefficient between CEI and C_trough_ was −0.425 (*p* = 0.034), while excluding the effects of albumin and the FIB-4 index. Receiver operating characteristic (ROC) curve analysis showed that the CEI was relatively accurate in predicting hyperbilirubinemia, with area under the ROC of 0.882. Multivariate analysis showed that the CEI < 1.61 was the only independent predictor related to the development of hyperbilirubinemia, with an odds ratio of 9.08 (95% confidence interval 1.05–78.86, *p* = 0.046).

**Conclusions:**

Hepatic enhancement with gadoxetic acid was independently related to paritaprevir concentration and was an independent pretreatment factor in predicting hyperbilirubinemia. Gadoxetic acid–enhanced MR imaging can therefore be useful in determining the risk of paritaprevir-induced hyperbilirubinemia.

## Introduction

Hepatitis C virus (HCV) is one of the main causes of chronic liver disease, affecting 160–180 million people worldwide [1, 2]. In Japan, about 2 million people are infected with HCV, and HCV genotypes 1b, 2a, and 2b are responsible for approximately 70%, 20%, and 10% of the infections, respectively [3].

The regimen of ombitasvir, paritaprevir, and ritonavir (OBV/PTV/r) without or with ribavirin has been available in Japan since 2015 and 2016, respectively [4, 5]. Ritonavir is included for its booster effect on paritaprevir by cytochrome P450 3A inhibitor. Paritaprevir, a nonstructural (NS)3/4A protease inhibitor, is mainly eliminated via hepatobiliary excretion. It is carried into the hepatocyte by hepatic transporters including organic anion–transporting polypeptides (OATP)1B1 and OATP1B3, located at the sinusoidal membranes of hepatocytes. These transporters also carry unconjugated bilirubin [6, 7]. Subsequently, paritaprevir is eliminated via breast cancer resistance protein, located at the canalicular membranes of hepatocytes [7–9]. During treatment, however, a mild to moderate increase in serum bilirubin concentration, which is related to the inhibition of bilirubin transporters, has been observed in some cases [5, 6, 8, 10]. Clinically, in patients receiving paritaprevir, 23% had elevation in total bilirubin (≥ 2.25 mg/dl), mainly indirect (unconjugated) bilirubin levels, without elevations in liver enzymes during the regimen of paritaprevir/ritonavir, ombitasvir, and dasabuvir with or without ribavirin [10].

Various direct antiviral agent (DAA) regimens are rapidly evolving for patients with chronic HCV infection, and serological viral response has progressed [11]. Safety is the other necessary element in anti-HCV therapy. Not all patients are able to tolerate the treatment because of adverse events. Potential drug–bilirubin interaction, one type of adverse event during therapy, can occur with NS3/4A protease inhibitors such as paritaprevir. This issue remains an important aspect of management in DAA therapy [12]. Paritaprevir-induced hyperbilirubinemia is presumably caused by the inhibitory effect of the drug on OATP1B1/1B3 [10]. Nevertheless, there are few reports describing these interactions in clinical practice and the relationship between the plasma concentration and the development of hyperbilirubinemia.

Interindividual variability in the gene encoding OATP transporters can lead to interindividual differences in pharmacokinetics. Gadoxetic acid (gadolinium-ethoxybenzyl-diethylenetriamine pentaacetic acid, Gd-EOB-DTPA), a liver-specific magnetic resonance (MR) contrast agent, is also a substrate of OATP1B1 and OATP1B3 [13]. Recent studies have suggested that enhancement of background liver by Gd-EOB-DTPA may be influenced not only by liver function but also by polymorphisms in the genes encoding OATPs [14, 15].

We therefore hypothesized that quantitative assessment of hepatic parenchymal enhancement by Gd-EOB-DTPA in MR imaging could predict the pharmacokinetics of paritaprevir and may anticipate the development of hyperbilirubinemia. To test this hypothesis, we investigated hepatic enhancement by Gd-EOB-DTPA, plasma paritaprevir concentration, and bilirubin increase in patients with hepatitis C who had undergone treatment with the regimen of OBV/PTV/r.

## Materials and methods

### Patients and treatments

This was a single-center, exploratory, prospective study. The study was approved by both the ethical review board of Juntendo University Faculty of Medicine (Jundai-Irin No-2016135) and the Ethics Committee for Human Genome/Gene Analysis Research at Kanazawa University Graduate School of Medical Sciences (No. 2016–004, serial No. 432) and was carried out in accordance with the 1964 Declaration of Helsinki and its later amendments. The study was also registered with UMIN clinical trials as UMIN000022587. Written informed consent was obtained from all patients before enrollment. The study enrolled adult patients with HCV genotype 1b chronic hepatitis or compensated liver cirrhosis using OBV/PTV/r (VIEKIRAX^®^; AbbVie, Inc., North Chicago, Illinois, USA) at Juntendo University Nerima Hospital. Analysis of resistance-associated variations of the NS5A Y93H substitutions in the baseline sequence was performed in a commercial laboratory (SRL, Inc., Tokyo, Japan), and the patients with the mutant type of NS5A Y93H were excluded. Patients with renal dysfunction (estimated glomerular filtration rate < 40 ml/min/1.73 m^2^) were excluded. A precaution in OBV/PTV/r treatment is coadministration of statin medications. Patients taking simvastatin and atorvastatin were excluded; no other patients were taking any other type of statin. Consequently, this study involved 27 consecutive Japanese patients (8 males and 19 females), with ages ranging from 30 to 83 years, treated between June 2016 and July 2017. Twenty patients with chronic hepatitis and seven patients with liver cirrhosis were included. All patients were treated with OBV/PTV/r, at a dose of 25/150/100 mg, administered orally once daily.

Gd-EOB-DTPA–enhanced MR imaging was undertaken and laboratory data obtained within 2 weeks before starting treatment. In patients taking ursodeoxycholic acid, a substrate of OATP1B1 and OATP1B3 [16], the medication was discontinued before undergoing the MR imaging and starting the antiviral therapy. The FIB-4 index, a measure of liver fibrosis, was also determined before treatment [17]. Serum total and unconjugated bilirubin concentrations were monitored throughout the course of antiviral therapy, and toxicities related to hyperbilirubinemia were graded according to World Health Organization (WHO) toxicity grades. Hyperbilirubinemia was defined as a total bilirubin concentration ≥ 1.6 mg/dl (WHO Grade ≥ 2).

Fasting steady-state plasma trough concentrations (C_trough_) of paritaprevir were determined from blood samples obtained 7 d after the start of treatment. The patients were given the same breakfast of 338 kcal, with 15% of calories from protein, 60% from fat, and 25% from carbohydrates. After the meal, the patients took the drugs orally. Paritaprevir concentrations at 2, 4, and 6 h after dosing were determined. Blood samples were centrifuged within 30 min at 1,500 rpm for 10 min at 4°C, and plasma concentrations of paritaprevir were determined using liquid chromatography–tandem mass spectrometry, as described [18]. The area under the plasma concentration curve from 0 to 6 h (AUC_0–6 h_) of paritaprevir after dosing was calculated using the trapezoidal rule.

### MR analysis

All patients underwent MR imaging with the 1.5T Signa Excite ^™^ HD system (GE Healthcare, Waukesha, WI, USA) and an eight-channel, phased-array body coil. The section thickness was 2.2 mm. The imaging protocol consisted of T1-weighted, fat-suppressed 3D gradient-recalled echo sequences using parallel imaging with phased-array uniformity enhancement (repetition time 4.0 ms; echo time 1.9 ms; flip angle 15°; matrix 352 × 224; field of view 400 × 360 mm; acquisition time 19 s).

After obtaining precontrast scans, Gd-EOB-DTPA (0.1 ml/kg body weight; EOB Primovist^™^/Eovist^®^ injection; Bayer HealthCare, LLC, Whippany, New Jersey, USA) was given intravenously, as a bolus at a rate of 3 ml/s. At 20 min after administration, hepatobiliary images were taken in the transverse plane and quantified using a contrast enhancement index (CEI) [19, 20], with the number of measurement areas modified [21]. Briefly, precontrast and hepatobiliary MR images were displayed using the FuncTool^™^ software (GE Healthcare) accompanying the imager, and 100- to 150-mm^2^ regions of interest (ROIs) of the bilateral major psoas muscles and 12 points of the liver excluding large vessels, hepatic cysts, and prominent artifacts were selected [22]. The ROIs were identified by a combination of Couinaud’s segments (S1–S8) and zonal locations (central and peripheral) as follows: S1, central; S2, central; S2, peripheral; S3, central; S3, peripheral; S4, central; S4, peripheral; S5, central; S6/7, central; S6, peripheral; S7, peripheral; and S8, peripheral [21]. The liver to major psoas muscle ratios (LMRs), based on average signal intensities (SIs), were determined before (SI_pre_) and at 20 min (SI_20_) after Gd-EOB-DTPA administration. ROIs of the same shape and size were placed at the same points on axial images before and after the administration, and CEI was calculated as LMR_20_/LMR_pre_. Analyses were independently made by two radiologists (Y.O. and M.A.), who were not authors and had more than 15 years of experience each in liver MR imaging. The average value of all SIs measured by the two radiologists were used for quantitative analysis of the CEI.

### Statistical analysis

Pearson correlations were performed to determine the associations between pairs of variables, after the data were tested for normal distribution by the Shapiro–Wilk normality test. Because of the nonnormality of the paritaprevir C_trough_, natural logarithmic transformation was used in the analysis to satisfy the requirement for normality. The independent contribution of each variable to the CEI was determined by calculating the partial correlation coefficient between CEI and C_trough_, while excluding the effects of serum albumin and the FIB-4 index. Differences in continuous variables were compared with the paired t-test after the data were tested for normal distribution by the Shapiro–Wilk normality test. Fisher’s exact test was used to compare categorical data. These statistical analyses were performed using SPSS Statistics for Windows, Version 22 (IBM Corp., Tokyo, Japan). The cut-off value of CEI for the prediction of hyperbilirubinemia was calculated using a receiver operating characteristic (ROC) curve. Variables of pretreatment with *p* values < 0.25 in the univariate analysis were re-evaluated by multiple logistic regression analysis to identify the factors associated with the development of hyperbilirubinemia. We evaluated the predictive performance of the analysis by assessing fit index by the use of Akaike’s Information Criterion and area under the ROC (AUROC) curve. All AUROCs were presented with 95% confidence intervals (CIs). These statistical analyses were performed using StatFlex, Version 6 (Artech Co., Ltd., Osaka, Japan). All tests were two-sided, and *p* values < 0.05 were considered statistically significant.

## Results

### Baseline characteristics and bilirubin increase during therapy

A total of 27 Japanese patients were enrolled in the study. The baseline characteristics of all patents included in this study are shown in Table 1. Their baseline median total bilirubin concentration was 0.6 mg/dl (range 0.4–1.4 mg/dl).

**Table 1:** Patient characteristics.

Characteristic	N = 27
Male/female	8/19
Age median, years (range)	75 (30–83)
Weight median, kg (range)	53 (29–100)
Chronic hepatitis/liver cirrhosis	20/7
Total bilirubin median, mg/dl (range)	0.6 (0.4–1.4)
Indirect bilirubin median, mg/dl (range)	0.4 (0.1–1.0)
Albumin median, g/dl (range)	4.1 (3.5–5.0)
Prothrombin time median, INR (range)	1.05 (0.93–1.2)
Platelets median, x 10^8^/L (range)	15.9 (6.6–27.0)
Alanine aminotransferase median, IU/L (range)	35 (14–126)
FIB-4 index median (range)	3.24 (0.64–10.96)

**Fig 1:**
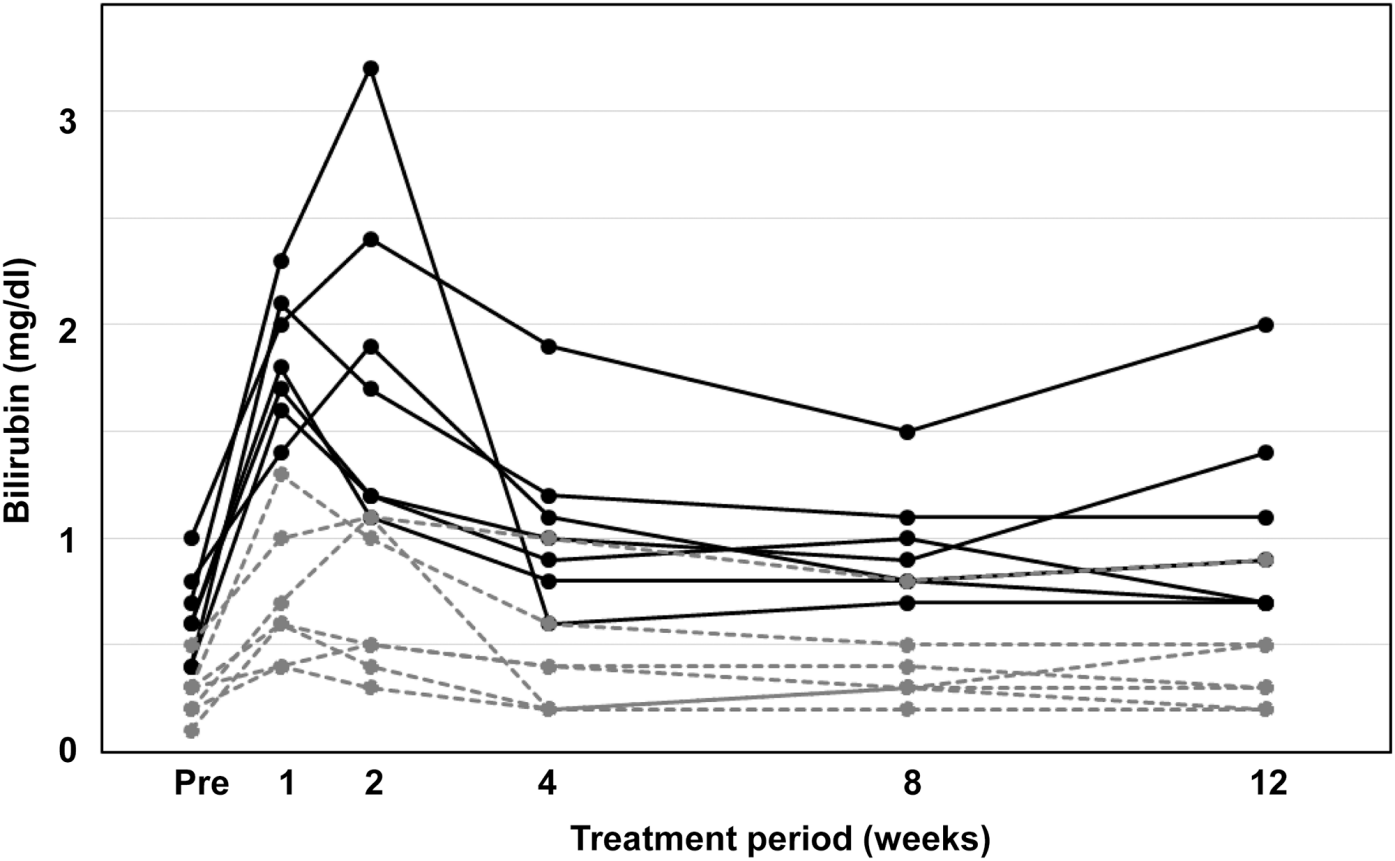
Individual time course of serum bilirubin concentration in seven patients treated with paritaprevir who developed hyperbilirubinemia (≥ 1.6 mg/dl). Black line, total bilirubin; gray dotted line, direct bilirubin.

During the 12-week course of treatment, seven patients (26%) had total bilirubin concentrations ≥ 1.6 mg/dl, equivalent to WHO toxicity Grade ≥ 2 (Fig 1). Of these seven patients, one had a maximum total bilirubin concentration of 3.2 mg/dl, followed by 2.4, 2.1, 1.9, 1.8, 1.7, and 1.6 mg/dl, equivalent to Grades 2–3. Their unconjugated bilirubin concentrations were 2.1, 1.3, 0.8, 1.4, 1.4, 1.1, and 1.3 mg/dl, respectively.

The seven patients showed an improvement in bilirubin levels without cessation of the antiviral regimen or need for decreased dosage. None of the patients had concomitant abnormalities in aminotransferase levels.

### Pharmacokinetic study and relationships between pharmacokinetic parameters of paritaprevir and liver function variables

**Fig 2:**
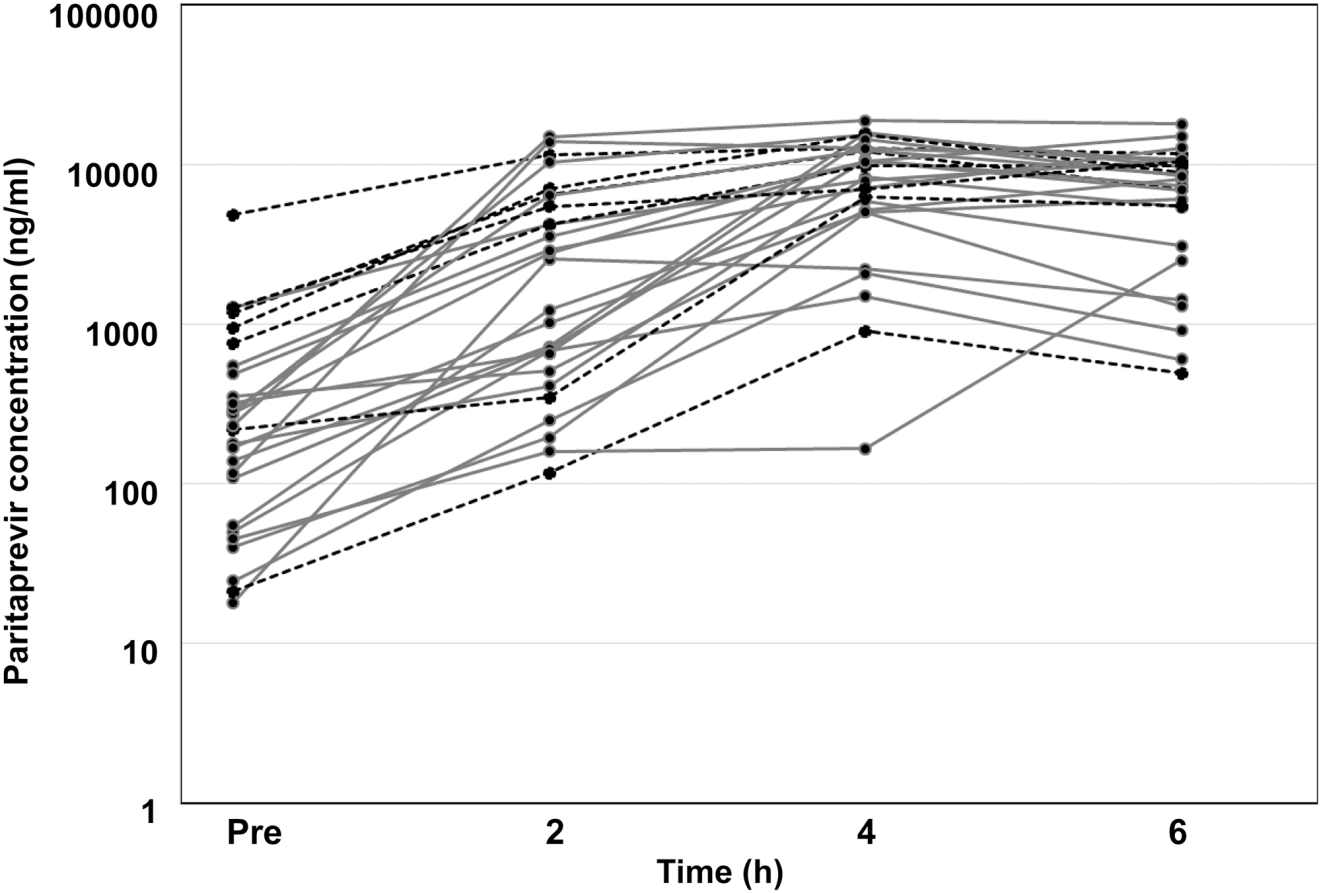
Time profile of plasma paritaprevir concentration 7 d after the start of treatment in each patient (trough concentration, concentration at 2 h, 4 h, and 6 h after dosing). Gray line, patients without hyperbilirubinemia (N = 20); black dotted line, patients with hyperbilirubinemia (N = 7).

**Fig 3:**
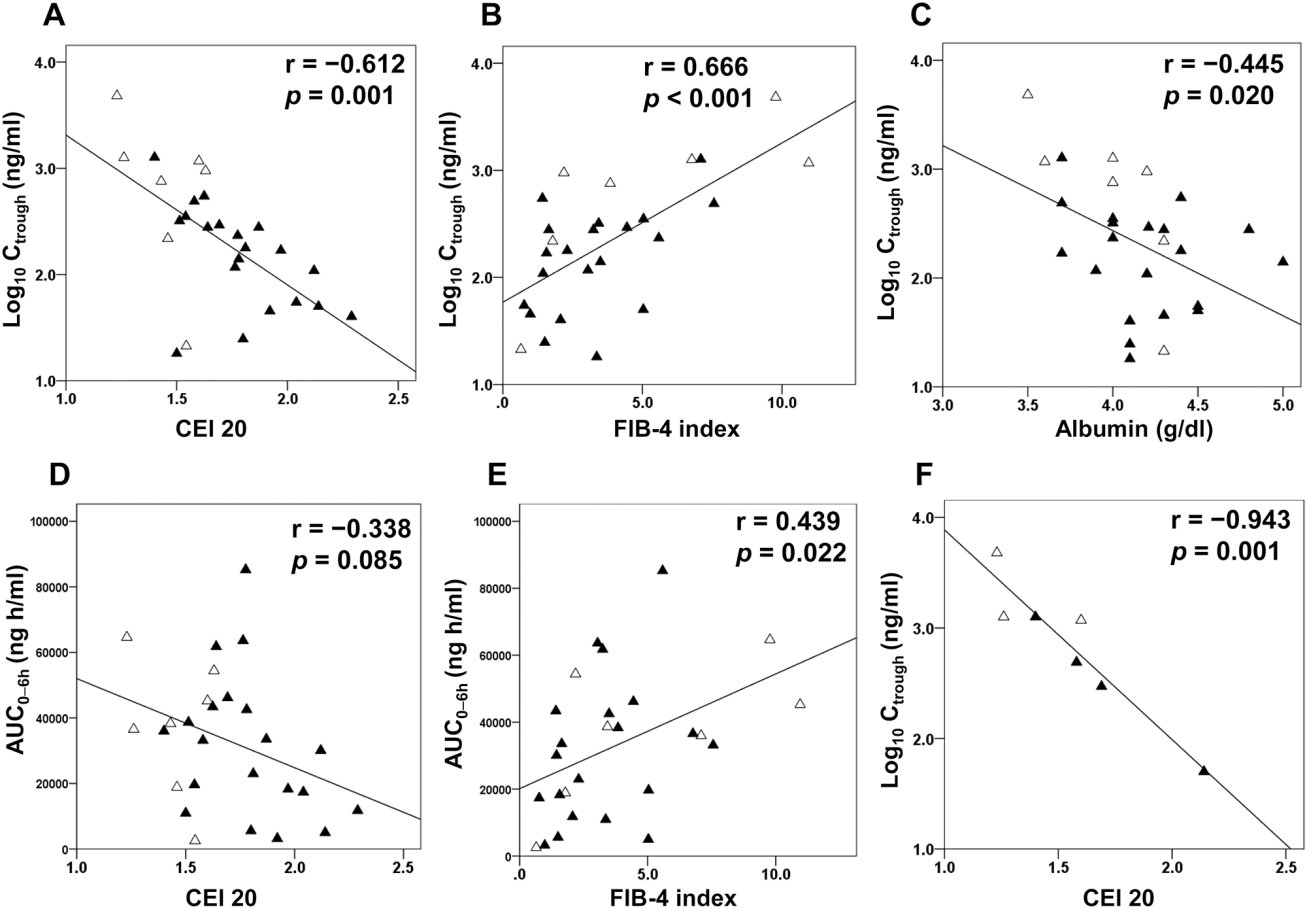
Relationship between pharmacokinetic parameters of paritaprevir and CEI 20 (A, D); FIB-4 index (B, E); and serum albumin concentration (C) in all patients and relationship between trough concentration of paritaprevir and CEI 20 in cirrhotic patients (F). Black triangle, patients without hyperbilirubinemia; open triangle, patients with hyperbilirubinemia. AUC_0–6h_, area under the blood concentration–time curve 0–6 h; CEI 20, contrast enhancement index at 20 min; C_trough_, trough concentration.

The pharmacokinetic studies of paritaprevir plasma concentration 7 d after the start of treatment are shown in Fig 2. Subsequently, as illustrated in Fig 3, the C_trough_ of paritaprevir showed significant negative correlations with CEI (r = −0.612, *p* = 0.001), and serum albumin concentration (r = −0.445; *p* = 0.020), as well as a significant positive correlation with the FIB-4 index (r = 0.666; *p* < 0.001). The AUC_0–6 h_ showed significant positive correlation with the FIB-4 index (r = 0.439; *p* = 0.022). In contrast, there was not a significantly weak inverse relationship between AUC_0–6 h_ and CEI (r = −0.338, *p* = 0.085). Only in cirrhotic patients as a subanalysis, the C_trough_ of paritaprevir showed significant negative correlations with CEI (r = −0.943, *p* = 0.001). Correlation coefficients and partial correlation coefficients between the C_trough_ of paritaprevir and variables such as CEI, albumin, and the FIB-4 index are shown in Table 2. The partial correlation coefficient between CEI and C_trough_ was −0.425 (*p* = 0.034).

**Table 2:** Correlation coefficients and partial correlation coefficients between variables.

	**Alb**	**FIB-4 Index**	**CEI 20**	**Log**_**10**_ **C**_**trough**_
**Alb**	--	−.583*	.361	−.445**
**FIB-4 index**	−.418*	--	−.503**	.666**
**CEI 20**	−.064	−.120	--	−.612**
**Log**_**10**_ **C**_**trough**_	−.057	0.452*	−.425*	--

Alb, albumin; CEI 20, contrast enhancement index at 20 min; C_trough_, trough concentration (of paritaprevir).

The upper right shows correlation coefficients, and lower left shows partial correlation coefficients.

* *p* < 0.05,

** *p* < 0.01.

### Variables associated with the development of hyperbilirubinemia and ROC curve analysis

As shown in Table 3, the C_trough_ of paritaprevir was significantly higher in patients with hyperbilirubinemia (*p* = 0.022) compared with those without hyperbilirubinemia, while there was no significant difference in the AUC_0–6 h_ of paritaprevir between the two groups (*p* = 0.278). In terms of pretreatment parameters, the CEI was significantly lower in patients with hyperbilirubinemia (*p* = 0.002) compared with those without hyperbilirubinemia. In contrast, there were no significant differences between these two groups in serum albumin level (*p* = 0.173), indirect bilirubin (*p* = 0.173), or the FIB-4 index (*p* = 0.278).

**Table 3:** Variables in patients without and with hyperbilirubinemia before and during treatment.

Variable	Without hyperbilirubinemia (N = 20)	With hyperbilirubinemia (N = 7)	*p* value
**Pretreatment**			
Age, years	68 ± 13	72 ± 19	0.556
Weight, kg	53.8 ± 15.4	51.9 ± 8.4	0.749
CH/LC	16/4	4/3	0.328
Total bilirubin, mg/dl	0.74 ± 0.32	0.64 ± 0.21	0.461
Indirect bilirubin, mg/dl	0.49 ± 0.25	0.37 ± 0.15	0.173
Albumin, g/dl	4.2 ± 0.35	4.0 ± 0.33	0.173
Prothrombin time, INR	1.05 ± 0.075	1.05 ± 0.065	0.510
Platelets, x 10^8^/L	16 ± 5.7	16 ± 6.5	0.881
ALT, IU/L	44 ± 29	46 ± 23	0.846
FIB-4 index	3.25 ± 1.99	5.14 ± 4.08	0.278
CEI 20	1.788 ± 0.238	1.451 ± 0.157	0.002
**During treatment**			
Log_10_ C_trough_, ng/ml	2.169 ± 0.484	2.768 ± 0.747	0.022
AUC, ng h/ml	31441 ± 21727	37224 ± 21015	0.278

ALT, alanine aminotransferase; AUC, area under the curve; CEI 20, contrast enhancement index at 20 min; CH, chronic hepatitis; C_trough_, trough concentration (of paritaprevir); LC, liver cirrhosis.

### ROC curve analysis

An ROC curve analysis was generated and the area under the curve (and its 95% CI) calculated for predicting the development of hyperbilirubinemia (Table 4). ROC analysis revealed that CEI < 1.61, serum albumin ≤ 4.2 g/dl, and indirect bilirubin ≥ 0.5 mg/dl at baseline were the best cutoff values to predict the development of paritaprevir-induced hyperbilirubinemia. Specifically, the CEI was highly accurate in predicting hyperbilirubinemia (AUROC 0.882, 95% CI 0.754–1.000, sensitivity 71.4%, specificity 75.0%, positive predictive value 50.0%, and negative predictive value 88.2%).

**Table 4:** ROC curve analysis of each baseline parameter and prediction of hyperbilirubinemia.

	Cut-off Value	Sensitivity, %	Specificity, %	AUROC (95% CI)	PPV, %	NPV, %
CEI 20	1.61	71.4	75.0	0.882 (0.754–1.000)	50.0	88.2
Alb, g/dl	4.2	57.1	55.0	0.621 (0.341–0.901)	30.8	78.6
Indirect bilirubin, mg/dl	0.5	42.9	65.0	0.568 (0.332–0.804)	30.0	76.5

Alb, albumin; AUROC, area under the receiver operating characteristic curve; CEI 20, contrast enhancement index at 20 min; NPV, negative predictive value; PPV, positive predictive value.

### Multivariate analysis

According to multivariate regression analysis including CEI, indirect bilirubin, and serum albumin, the CEI was a significant and independent pretreatment predictor associated with developing hyperbilirubinemia (odds ratio 9.08, *p* = 0.046, 95% CI: 1.05–78.86) with Akaike’s Information Criterion of 33.67 and AUROC of 0.739 (Table 5).

**Table 5:** Multivariate regression analysis predicting hyperbilirubinemia.

	Odds Ratio (95% CI)	*p* value
CEI 20 < 1.61	9.08 (1.05–78.86)	0.046
Indirect bilirubin ≥ 0.5 (mg/dl)	2.01 (0.26–15.37)	0.672
Alb ≤ 4.0 (g/dl)	0.84 (0.10–6.82)	0.162

Alb, albumin; CEI 20, contrast enhancement index at 20 min; CI, confidence interval.

## Discussion

This study indicated that liver parenchymal enhancement at 20 min of Gd-EOB-DTPA–enhanced MR imaging was independently predictive of plasma paritaprevir C_trough_ and the development of hyperbilirubinemia in patients with HCV treated with OBV/PTV/r.

Paritaprevir is a more potent in vitro inhibitor of OATP1B1, which is primarily responsible for the hepatic uptake of unconjugated bilirubin [23]. We have to be cautious regarding the development of hyperbilirubinemia by drug–bilirubin interactions. Gd-EOB-DTPA–enhanced MR imaging is a promising noninvasive tool in the quantification of liver fibrosis and preoperative hepatic function [19, 20, 24]. We therefore used MR imaging at the pretreatment stage to predict the intrahepatic accumulation of paritaprevir through quantitative analysis of parenchymal enhancement at the hepatobiliary phase. As expected, we found that the enhancement effect significantly correlated with paritaprevir plasma C_trough_. This might be because of the properties of Gd-EOB-DTPA, which is believed to be specifically taken up into hepatocytes by hepatic transporters such as OATP1B1 and OATP1B3 [13]. A previous clinical study indicated that the expression of OATP1B1, extracted from liver biopsy specimens, is decreased in accordance with the progression of chronic hepatitis C [25]. Liver impairment, genetic polymorphisms, and drug–drug interactions can alter the function of OATPs in patients with chronic liver disorders [15, 26]. Enhancement of background liver by Gd-EOB-DTPA may be influenced not only by liver function but by polymorphisms in the genes encoding OATPs [14, 15]. Specifically, our previous study clearly demonstrated that, independently of the degree of liver impairment, the SLCO1B1*1B haplotype and *SLCO1B3* 334T>G polymorphism influenced the enhancement of Gd-EOB-DTPA [15]. Unconjugated bilirubin is taken up by hepatocytes via several OATPs [23, 27]. Similarly, paritaprevir was shown, at least in vitro, to be a substrate of OATP1B1 and OATP1B3 [7, 28]. Therefore, quantitative analysis of Gd-EOB-DTPA–enhanced MR imaging potentially reflects comprehensive transporter imaging. This imaging may indicate decreased expression of OATPs resulting from liver impairment and interindividual variability in OATP function by their single nucleotide polymorphisms. Thus, the imaging can indirectly reflect paritaprevir exposure and anticipate the development of paritaprevir-induced hyperbilirubinemia.

Recently, we showed that the liver enhancement effect at the hepatobiliary phase of Gd-EOB-DTPA–enhanced MR imaging was related to the concentration of simeprevir, an NS3/4A protease inhibitor. We could predict the onset of hyperbilirubinemia during simeprevir and pegylated interferon plus ribavirin therapy [22]. However, that was a pilot study comprising 11 patients. As expected, the results of the current study, drawn from a more substantial cohort of 27 individuals, shows that comprehensive transporter imaging using Gd-EOB-DTPA could predict paritaprevir concentration and the development of hyperbilirubinemia because of drug–bilirubin interactions.

The ROC curve and multivariate analyses indicated that the best cut-off value for CEI at 20 min was 1.61, and the CEI was an independent pretreatment factor for predicting paritaprevir-induced hyperbilirubinemia. These findings enabled discrimination of HCV patients at risk for developing hyperbilirubinemia. CEI also correlated dependently with the C_trough_ of paritaprevir, which could explain the fact that the hepatobiliary phase of Gd-EOB-DTPA–enhanced MR imaging reflects OATP function, indicating uptake capability through the hepatocyte membrane. Nevertheless, biliary excretion has already occurred at the 20-min hepatobiliary phase [29], the degree of precision may also be lower if hepatic enhancement is measured at much later time points. Remarkable biliary excretion had already occurred at the 20-min hepatobiliary phase in one patient with a CEI of 1.5 at 20 minutes. This patient did not have hyperbilirubinemia and appears as an outlier in Fig 3a.

Gopalakrishnan et al. have shown that the range of paritaprevir exposure was comparable between Japanese and western patients [30]. However, these exposures were normally distributed in westerners. In contrast, those in the Japanese patients varied widely among subjects. There are large interindividual differences in pharmacokinetic profiles in Japanese people [30]. Tomita et al. indicated that the ethnic variability in the plasma exposure of statins (also substrates for OATP1B1), cannot be explained only by the difference in the allele frequencies of OATP1B1; the intrinsic ethnic variability in the activity of OATP1B1 must be considered [31]. Therefore, Gd-EOB-DTPA–enhanced MR imaging, a method that reflects the activity and function of OATPs caused by gene polymorphism, may be much more useful in the assessment of exposure to an NS3/4A protease inhibitor than analysis of gene polymorphisms alone.

Recent studies have addressed the unexpectedly high rate and pattern of recurrence of hepatocellular carcinoma (HCC) after HCV eradication by DAAs [32, 33]. HCC recurrence should be ruled out prior to anti-HCV therapy. Gd-EOB-DTPA–enhanced MR imaging is the most useful imaging technique for evaluating the presence or absence of small HCCs, including early HCC in patients with chronic liver disease [34, 35]. In fact, the imaging obtained before anti-HCV therapy can really be a “one-stop shop” for the exclusion of HCC and the predicted response to an NS3/4A protease inhibitor such as paritaprevir.

To our knowledge, this is the first substantial study to examine the association between quantitative analysis of hepatic enhancement by Gd-EOB-DTPA–enhanced MR imaging and the prediction of NS3/4A inhibitor–induced hyperbilirubinemia. However, gadoxetic acid–enhanced MR imaging should not necessarily be performed in all patients for the sake of institutional convenience. It might be reasonable that only patients with cirrhosis should undergo such imaging; these patients have increased paritaprevir exposure from impaired drug clearance, which can lead to a severe clinical condition such as drug-induced liver injury [36]. Our subanalysis only included patients with cirrhosis; the strong negative correlations between enhancement effect and paritaprevir C_trough_, also showed that the quantitative analysis of Gd-EOB-DTPA–enhanced MR imaging would be well suited to clinical practice. In addition, this study could be applicable to predicting the response to plasma exposure to an anticancer drug that is a substrate for OATP1B1/1B3. Examples are SN-38, an active metabolite of irinotecan hydrochloride [37, 38] and paclitaxel [39]. Furthermore, the imaging technique could be used to identify adverse events related to the blood concentration of these drugs.

Our study has several limitations. First, in our cohort of 27 patients, we failed to show the impact of polymorphisms of OATP-related genes on paritaprevir concentrations and the development of hyperbilirubinemia (data not shown). It should be noted that the hepatic enhancement effect by Gd-EOB-DTPA was associated with the plasma concentration of paritaprevir and prediction of hyperbilirubinemia. Second, a treatment change had not been required in our study patients. Nevertheless, patients whose Gd-EOB-DTPA–enhanced MR imaging indicates hypo-enhancement require special monitoring for hyperbilirubinemia. Third, our use of a method directly measuring hepatic SI on Gd-EOB-DTPA–enhanced MR images was not the most precise measurement technique. The optimal Gd-EOB-DTPA–associated method for estimating hepatic function and OATP activity has not been established [40]. If a more precise method for determining intrahepatic Gd concentration were established, comprehensive transporter imaging using Gd-EOB-DTPA–enhanced MR would be more accurate.

In conclusion, this study showed that liver parenchymal enhancement in Gd-EOB-DTPA–enhanced MR imaging performed at pretreatment was associated with the concentration of paritaprevir. The quantitative analysis of liver parenchyma in the hepatobiliary phase at 20 min after injection could represent a useful parameter in detecting paritaprevir-induced hyperbilirubinemia during anti-HCV therapy.
